# Duration of immunity following full vaccination against SARS-CoV-2: a systematic review

**DOI:** 10.1186/s13690-022-00935-x

**Published:** 2022-09-02

**Authors:** Isaac Yeboah Addo, Frederick Asankom Dadzie, Sylvester Reuben Okeke, Caleb Boadi, Elijah Frimpong Boadu

**Affiliations:** 1 Centre for Social Research in Health, University of New South Wales, Sydney, 2052 Australia; 2 Centre for Ecosystem Science, School of Biological, Earth and Environmental Sciences, University of New South Wales, Sydney, 2052 Australia; 3 Evolution and Ecology Research Centre, School of Biological, Earth and Environmental Sciences, University of New South Wales, Sydney, NSW 2052 Australia; 4 Department of Operations and Management Information Systems, University of Ghana, Accra, Ghana; 5 School of Built Environment, University of New South Wales, Sydney, 2052 Australia

**Keywords:** SARS-CoV-2, Waning, Immunity, Omicron, Booster, COVID-19 vaccines, Equity

## Abstract

**Background:**

As vaccine roll-out continues across the globe as part of the efforts to protect humanity against SARS-CoV-2, concerns are increasingly shifting to the duration of vaccine-induced immunity. Responses to these concerns are critical in determining if, when, and who will need booster doses following full vaccination against SARS-CoV-2. However, synthesised studies about the durability of vaccine-induced immunity against SARS-CoV-2 are scarce. This systematic review synthesised available global evidence on the duration of immunity following full vaccination against SARS-CoV-2.

**Methods:**

We searched through Psych Info, Web of Science, Scopus, Google Scholar, PubMed, and WHO COVID-19 databases for relevant studies published before December 2021. Five eligibility criteria were used in scrutinising studies for inclusion. The quality of the included studies was assessed based on Joana Briggs Institute’s (JBI) Critical Appraisal tool and Cochrane’s Risk of Bias tool—version 2 (RoB 2), while the reporting of the results was guided by the Synthesis Without Meta-analysis (SWiM) guidelines.

**Results:**

Twenty-seven out of the 666 identified studies met the inclusion criteria. The findings showed that vaccine-induced protection against SARS-CoV-2 infections builds rapidly after the first dose of vaccines and peaks within 4 to 42 days after the second dose, before waning begins in subsequent months, typically from 3 to 24 weeks. Vaccine-induced antibody response levels varied across different demographic and population characteristics and were higher in people who reported no underlying health conditions compared to those with immunosuppressed conditions.

**Conclusions:**

Waning of immunity against SARS-CoV-2 begins as early as the first month after full vaccination and this decline continues till the sixth month when the level of immunity may not be able to provide adequate protection against SARS-CoV-2. While the evidence synthesised in this review could effectively inform and shape vaccine policies regarding the administration of booster doses, more evidence, especially clinical trials, are still needed to ascertain, with greater precision, the exact duration of immunity offered by different vaccine types, across diverse population characteristics, and in different vulnerability parameters.

**Registration:**

The protocol for this review was pre-registered with the International Prospective Register of Systematic Reviews [PROSPERO] (Registration ID: CRD420212818).

## Background

The evolving COVID-19 pandemic remains a significant global health issue. All over the world, the impact of the pandemic continues to transcend health to affect social, cultural, religious, political, and economic activities. It has been more than two years into the pandemic and yet there is still cautious optimism regarding eradication, or at least, effective control of SARS-CoV-2, the pathogenic agent associated with the pandemic. Although safety concerns around rare side effects of the vaccines [1], breakthrough infections following full vaccination [2], the emergence of new variants of SARS-CoV-2 such as Delta and Omicron [3], inequitable distribution of vaccines [4], and conspiracy theories around the pandemic [5, 6], may have impacted the excitement that followed the development, approval, and roll-out of various vaccines, substantial global evidence indicates that full vaccination significantly reduces COVID-19-related hospitalisations and deaths [7, 8].

Considering that vaccination has become a “silver bullet” shaping political decisions and health responses to the current pandemic, there is a need for timely and continuous empirical knowledge to inform such policies and responses. Against this background, understanding the duration of vaccine-induced immunity is critical to vaccine policy formulation and review about if, when, and who needs a vaccine booster. A seemingly plausible argument for promoting vaccine equity is whether booster shots are justified when a considerable proportion of the global population does not have access to even the first dose [9]. Understanding the durability of vaccine-induced protection could serve a dual purpose of justifying boosters, especially for vulnerable populations as well as for promoting equitable distribution of vaccines, especially in settings where they are more critically needed instead of ‘administering’ boosters to people whose vaccine-induced protection is still strong.

While many reviews have been conducted to establish evidence around the effectiveness and safety of COVID-19 vaccines [10–12], systematic reviews synthesising evidence on the duration of vaccine-induced immunity are scarce. This systematic review synthesises the global evidence on the durability of immunity following full vaccination against SARS-CoV-2. Considering that different vaccines may provide different levels of effectiveness, we also assessed available evidence around the waning of immunity based on vaccine types and discussed the implications of this evidence for both booster doses and equitable distribution of vaccines.

## Methods

### Searches

This systematic review was conducted in line with the Preferred Reporting Items for Systematic Reviews and Meta-Analyses (PRISMA) guidelines [13] and was pre-registered with PROSPERO (Registration ID: CRD420212818). Two authors (IYA and FAD) conducted literature searches on Psych Info, Web of Science, Scopus, Google Scholar, PubMed, and WHO COVID-19 database for relevant studies on duration and waning of vaccine immunity following full vaccination. We defined “full vaccination” based on the Australian Technical Advisory Group on Immunisation (ATAGI) definition as at the time of the literature search [14]. Full vaccination was defined as having received two doses of any Therapeutic Goods Administration (TGA) or WHO-approved two-dosage COVID-19 vaccine at least 14 days apart, except for the Janssen (Johnson and Johnson) COVID-19 vaccine, where they are regarded as fully vaccinated 7 days after the single dose [14].

The following search term was used in the databases with word builders (e.g., PubMed): “((((waning immunity) OR (duration of immunity)) OR (period of immunity)) AND (SARS-CoV-2vaccines)) OR (SARS-CoV-2 vaccines)”. On the other hand, the following search term was used in databases without word builders (e.g., Google Scholar, and WHO COVID-19 database): “waning or duration of immunity following full SARS-CoV-2 vaccination”.

### Study inclusion and exclusion criteria

Studies were included irrespective of the age or sex of participants and irrespective of the health status of participants. There was no restriction to vaccine type so far as the vaccine was registered and approved by the WHO as of 1 June 2021 and there was no restriction to the method of antibody detection, i.e., studies that reported antibodies via blood, serum, saliva, or plasma testing were all considered. However, studies should meet all the following criteria: 1) should be clinical trials, longitudinal studies, case–control studies, or cohort studies; 2) should contain primary data; 3) should be published by December 2021; 4) should be published in English; 5) participants in the reported studies should have received full vaccination against SARS-COV-2 (i.e. all two doses for two-dosage vaccines or 1 dose for one-dosage vaccine depending on vaccine type). We excluded studies based on animal data.

### Definition of key terms

Waning of immunity was defined as the loss of protective antibodies over time following full vaccination against the SARS-CoV-2 virus or the reduction in the immune response to the SARS-CoV-2 virus following full vaccination [15, 16]. Duration of immunity was also defined as the time point at which vaccine-induced immunity begins to decline and provides less protection for a fully vaccinated person [17]. Vaccine-induced immunity was defined as immunity acquired through the introduction of a killed or weakened form of the disease organism through vaccination [18].

### Outcomes

Two main outcomes were assessed: effects of SARS-CoV-2 vaccines on immunity and the period of waning or duration of immunity following full vaccination against SARS-CoV-2.

### Study screening and selection

First, all duplicates and unrelated studies were removed from the search results using the “Find Duplicates” function in Endnote software. The titles and abstracts of the remaining studies were exported from Endnote to Microsoft Office Excel for easy screening. Next, the titles and abstracts of the remaining studies were independently scrutinised for eligibility against the inclusion and exclusion criteria by all five authors (IYA, FAD, SRO, CB, and EFB). Columns were created in Excel where included studies were marked green, excluded studies were marked red, and undecided studies were marked yellow. Another column was created to allow each reviewer the chance to give reasons for excluding ‘ineligible’ studies. The screening output for each author was combined and those with three or more ‘green marks’ were automatically selected for full-text review whereas those with three or more ‘red marks’ were automatically excluded. A list of the remaining studies was developed, and disagreements were resolved through group discussions. Following that, a full-text screening against the inclusion and exclusion criteria as well as the study objectives was conducted by all the authors for the remaining studies to ensure that the relevant studies were reserved. A new list of included studies prepared independently by all the authors was compared and differences that arose were resolved through discussion. A flow diagram is presented in Fig. 2 to show the results of the study search and selection processes.

### Study quality assessment

**Fig. 1:**
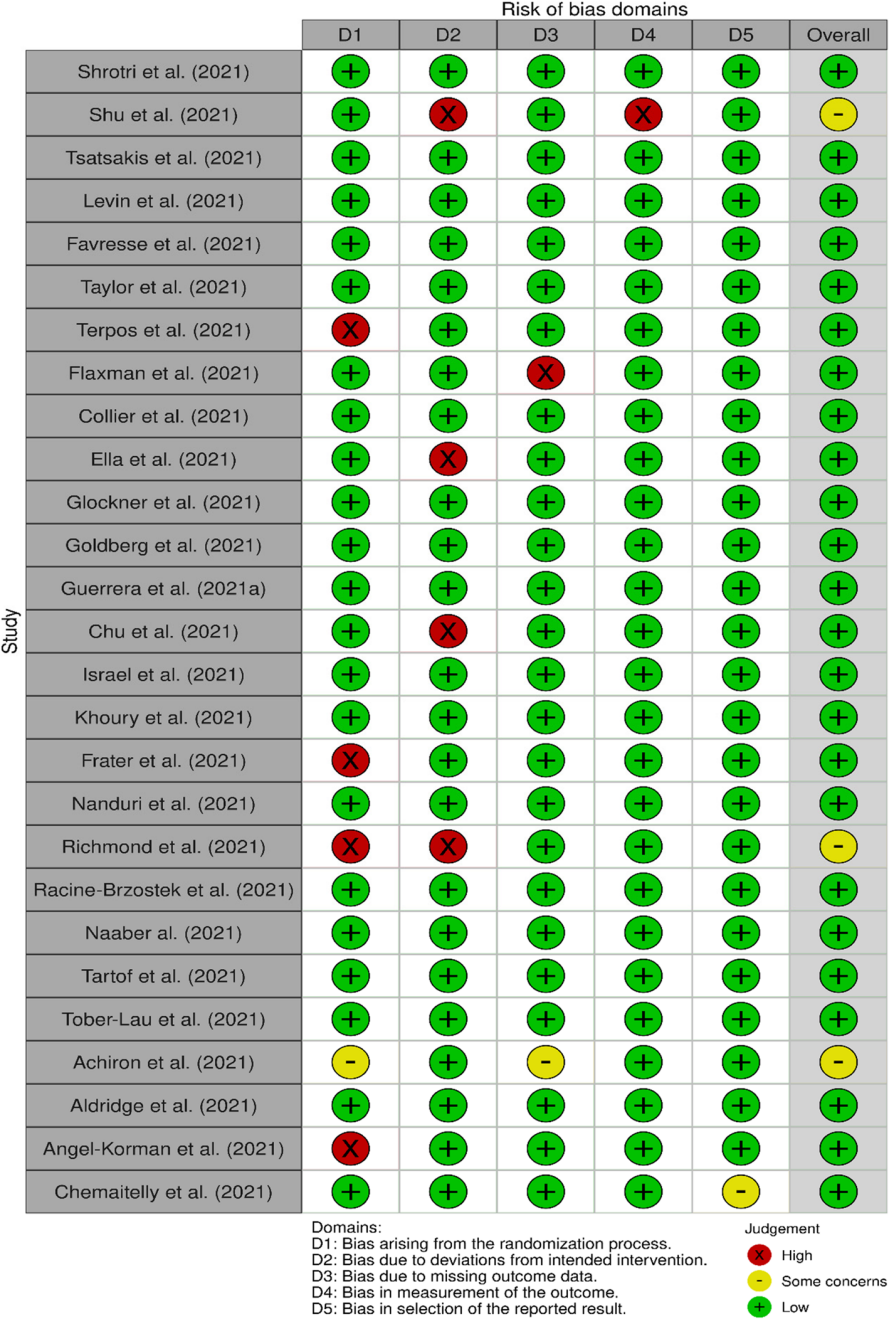
Results of Risk of Bias Assessment based on the Cochrane Risk of Bias tool—version 2 (RoB 2)

As shown in Table 1, the methodological quality of the included studies was assessed by all the authors using the Joanna Briggs Critical Appraisal checklist. We agreed to use the following response options to the questions shown in Table 1: “yes” or “no” or “unclear” or “not applicable”. When three (3) out of the five (5) reviewers recommended that a study should be included in the review after providing their independent yes and no responses, the study in question was automatically included in the review. In cases where less than 3 reviewers recommended that we include a study, we resolved the discrepancies through discussion and took a final decision to include or exclude through voting. Following that, the risk of bias was assessed by four of the authors (FAD, SRO, CB, and EFB) using the Cochrane Risk of Bias tool—version 2 (RoB 2) as illustrated in Fig. 1 [19]. Bias was assessed with the following domains: bias arising from the randomisation process, bias due to deviations from the intended interventions, bias due to missing outcome data, bias in the measurement of outcomes, and bias arising from selective reporting of results [19]. For robust and unbiased analysis, authors were blinded to each other’s assessment and the results were compared after everyone completed their review. Any disagreement was discussed with the first author (IYA).

**Table 1:** Results after quality appraisal using the Joana Briggs Institute’s (JBI) critical appraisal tool

**S/n**	**Study**	*Were the criteria for inclusion in the sample clearly defined?*	*Were the study subjects and the setting described in detail?*	*Was the exposure measured in a valid and reliable way?*	*Were objective and standard criteria used for measurement of the condition?*	*Were confounding factors identified?*	*Were strategies to deal with confounding factors stated?*	*Were the outcomes measured in a valid and reliable way?*	*Was appropriate statistical analysis used?*	*Decision*
1	Shrotri et al. (2021) [17]	Yes	Yes	Yes	Yes	Yes	Unclear	Yes	Yes	Include
2	Shu et al. (2021) [20]	Yes	Yes	Unclear	Yes	Yes	Unclear	Yes	Unclear	Include
3	Tsatsakis et al. (2021) [15]	Yes	Yes	Yes	Yes	Yes	Unclear	Yes	Yes	Include
4	Levin et al. (2021) [21]	Yes	Yes	Yes	Yes	Yes	Unclear	Yes	Yes	Include
5	Favresse et al. (2021) [22]	Yes	Yes	Yes	Yes	Yes	Unclear	Yes	Yes	Include
6	Taylor et al. (2021) [23]	Yes	Yes	Yes	Yes	Yes	Unclear	Yes	Yes	Include
7	Terpos et al. (2021) [24]	Yes	Yes	Yes	Yes	Yes	Unclear	Yes	Unclear	Include
8	Flaxman et al. (2021) [25]	Yes	Unclear	Yes	Yes	Yes	Unclear	Yes	Yes	Include
9	Collier et al. (2021) [26]	Yes	Yes	Yes	Yes	Yes	Unclear	Yes	Yes	Include
10	Ella et al. (2021) [27]	Unclear	Yes	Yes	Yes	Yes	Unclear	Yes	Yes	Include
11	Glockner et al. (2021) [28]	Yes	Yes	Yes	Yes	Yes	Unclear	Yes	Yes	Include
12	Goldberg et al. (2021) [29]	Yes	Yes	Yes	Yes	Yes	Unclear	Yes	Yes	Include
13	Guerrera et al. (2021) [30]	Yes	Yes	Yes	Yes	Yes	Unclear	Yes	Yes	Include
14	Chu et al. (2021) [31]	Yes	Unclear	Yes	Yes	Yes	Unclear	Yes	Yes	Include
15	Israel et al. (2021) [32]	Yes	Yes	Yes	Yes	Yes	Unclear	Yes	Yes	Include
16	Khoury et al. (2021) [33]	Yes	Yes	Yes	Yes	Yes	Unclear	Yes	Yes	Include
17	Frater et al. (2021) [34]	Unclear	Yes	Yes	Yes	Yes	Unclear	Yes	Yes	Include
18	Nanduri et al. (2021) [35]	Yes	Yes	Yes	Yes	Yes	Unclear	Yes	Yes	Include
19	Richmond et al. (2021) [36]	Unclear	Yes	Unclear	Yes	Yes	Unclear	Yes	Yes	Include
20	Racine-Brzostek et al. (2021) [37]	Yes	Yes	Yes	Yes	Yes	Unclear	Yes	Yes	Include
21	Naaber et al. (2021) [38]	Yes	Yes	Yes	Yes	Yes	Unclear	Yes	Yes	Include
22	Tartof et al. (2021) [39]	Yes	Yes	Yes	Yes	Yes	Unclear	Yes	Yes	Include
23	Tober-Lau et al. (2021) [40]	Yes	Yes	Yes	Yes	Yes	Unclear	Yes	Yes	Include
24	Achiron et al. (2021) [41]	Unclear	Yes	Yes	Yes	Yes	Unclear	Yes	Yes	Include
25	Aldridge et al. (2021) [42]	Yes	Yes	Yes	Yes	Yes	Unclear	Yes	Yes	Include
26	Angel-Korman et al. (2021) [43]	Yes	Unclear	Yes	Yes	Yes	Unclear	Yes	Yes	Include
27	Chemaitelly et al. (2021) [44]	Yes	Yes	Yes	Yes	Yes	Unclear	Yes	Yes	Include

Response options: “yes” or “no” or “unclear” or “not applicable

### Data extraction strategy

A template was developed in Microsoft Office Word containing various relevant thematic areas: authors and publication date, study design, country of study, type of vaccine, research participants, number of participants, vaccine effects on immunity, duration of immunity following full vaccination, limitations of the study, and key conclusions of the study. Data were extracted independently by four of the authors (IYA, FAD, SRO, and EFB) and the extracted data were examined independently by the remaining author (CB). Any disagreements that arose among the reviewers were consistently resolved through discussion. Summaries of the extracted data are presented in Table 2.

**Table 2:** Characteristics of included studies

S/n	Study	Suggested study type	Country	Type of vaccine	Research participants	Number of participants	Study Title
1	Shrotri et al. (2021) [17]	Cross-sectional	UK	BNT162b2 and ChAdOx1	Fully vaccinated adults	605	Spike-antibody waning after second dose of BNT162b2 or ChAdOx1
2	Shu et al. (2021) [20]	Randomised controlled trials	China	V-01	General population	880	Immunogenecity and safety of a recombinant fusion protein vaccine (V-01) against coronavirus disease 2019 in healthy adults: a randomised, double-blind, placebo-controlled, phase II trial
3	Tsatsakis et al. (2021) [15]	Prospective and interventional study	Greece	BNT162b2	Healthcare professionals	517	Immune response (IgG) following full inoculation with BNT162b2 COVID19 mRNA among healthcare professionals
4	Levin et al. (2021) [21]	Longitudinal prospective study	Israel	BNT162b2	Healthcare workers	4,868	Waning Immune Humoral Response to BNT162b2 Covid-19 Vaccine over 6 Months
5	Favresse et al. (2021) [22]	Prospective and interventional study	Belgium	BNT162b2	Healthcare professionals	200	Antibody titres decline 3-month post-vaccination with BNT162b2
6	Taylor et al. (2021) [23]	Longitudinal study/ Multiple blood sample tests	US	Ad26.COV2.S, BNT162b2 & mRNA-1273	SARS-COV-2 presumed positive samples	74	Semi-quantitative, high throughput analysis of SARS-CoV-2 neutralising antibodies: Measuring the level and duration of immune response antibodies post-infection/vaccination
7	Terpos et al. (2021) [24]	Randomised controlled trial	Greece	BNT162b2 & ChAdOx1	Patients with plasma cell neoplasms	276	The neutralising antibody response post-COVID-19 vaccination in patients with myeloma is highly dependent on the type of anti-myeloma treatment
8	Flaxman et al. (2021) [25]	Randomised controlled trial	UK	ChAdOx1 (AZD1222)	Volunteers	90	Reactogenicity and immunogenicity after a late second dose or a third dose of ChAdOx1 nCoV-19 in the UK: a substudy of two randomised controlled trials (COV001 and COV002)
9	Collier et al. (2021) [26]	Cohort study	England	BNT162b2	The elderly and younger healthcare workers	140	Age-related immune response heterogeneity to SARS-CoV-2 vaccine BNT162b2
10	Ella et al. (2021) [27]	Randomised controlled trial	India	BBV152 (Covaxin)	Healthy adults and adolescents	380	Safety and immunogenicity of an inactivated SARS-CoV-2 vaccine, BBV152: interim results from a double-blind, randomised, multicentre, phase 2 trial, and 3-month follow-up of a double-blind, randomised phase 1 trial
11	Glockner et al. (2021) [28]	Longitudinal study	Germany	BNT162b2, ChAdOx1 & mRNA-1273	Hospital staff	22	Robust Neutralising Antibody Levels Detected after Either SARS-CoV-2 Vaccination or One Year after Infection
12	Goldberg et al. (2021) [29]	Observational study	Israel	BNT162b2	General population	4,791,398	Waning Immunity after the BNT162b2 Vaccine in Israel
13	Guerrera et al. (2021) [30]	Longitudinal study	Italy	BNT162b2	Healthcare workers and scientists	71	BNT162b2 vaccination induces durable SARS-CoV-2 specific T cells with a stem cell memory phenotype
14	Chu et al. (2021) [31]	Randomised control trial	USA	mRNA-1273	Healthy adults	600	A preliminary report of a randomized controlled phase 2 trial of the safety and immunogenicity of mRNA-1273 SARS-CoV-2 vaccine
15	Israel et al. (2021) [32]	Retrospective cohort study	Israel	BNT162b2	General population	33,993	Elapsed time since BNT162b2 vaccine and risk of SARS-CoV-2 infection in a large cohort
16	Khoury et al. (2021) [33]	Longitudinal prospective study	Israel	BNT162b2	Healthcare personnel	100	COVID-19 Vaccine–Long Term Immune Decline and breakthrough infections
17	Frater et al. (2021) [34]	Randomised controlled trial	London, UK	ChAdOx1	People with HIV	54	Safety and immunogenicity of the ChAdOx1 nCoV-19 (AZD1222) vaccine against SARS-CoV-2 in HIV infection: a single-arm substudy of a phase 2/3 clinical trial
18	Nanduri et al. (2021) [35]	Observational study/longitudinal	USA	BNT162b2 & mRNA-1273	Elderly people in nursing homes	7,807,798	Effectiveness of Pfizer-BioNTech and Moderna Vaccines in Preventing SARS-CoV-2 Infection Among Nursing Home Residents Before and During Widespread Circulation of the SARS-CoV-2 B.1.617.2 (Delta) Variant—National Healthcare Safety Network, March 1-August 1, 2021
19	Richmond et al. (2021) [36]	Randomised controlled trial	Australia	SCB-2019	Adults and elderly	48	Persistence of the immune responses and cross-neutralizing activity with Variants of Concern following two doses of adjuvanted SCB-2019 COVID-19 vaccine
20	Racine-Brzostek et al. (2021) [37]	Longitudinal study	USA	BNT162b2	Vaccinated (previously diagnosed with SARS-COV-2 and never diagnosed) and unvaccinated individuals	350	More rapid, robust and sustainable antibody responses to mRNA COVID-19 vaccine in convalescent COVID-19 individuals
21	Naaber et al. (2021) [38]	Prospective study	Estonia	BNT162b2	Medical laboratory employees	122	Dynamics of antibody response to BNT162b2 vaccine after six months: a longitudinal prospective study
22	Tartof et al. (2021) [39]	Retrospective cohort study	USA	BNT162b2	Healthcare workers	3,436,957	Effectiveness of mRNA BNT162b2 COVID-19 vaccine up to 6 months in a large integrated health system in the USA: a retrospective cohort study
23	Tober-Lau et al. (2021) [40]	Prospective cohort study	Germany	BNT162b2	Older people and healthcare workers	177	Long-term immunogenicity of BNT162b2 vaccination in older people and younger health-care workers
24	Achiron et al. (2021) [41]	Prospective longitudinal cohort study	Israel	BNT162b2	Not found	39	Humoral SARS-COV-2 IgG decay within 6 months in COVID-19 healthy vaccinees: The need for a booster vaccine dose?
25	Aldridge et al. (2021) [42]	Nested longitudinal cohort	UK	BNT162b2 & ChAdOx1	Community based study (not found)	8,858	Waning of SARS-CoV-2 antibodies targeting the Spike protein in individuals post second dose of ChAdOx1 and BNT162b2 COVID-19 vaccines and risk of breakthrough infections: analysis of the Virus Watch community cohort
26	Angel-Korman et al. (2021) [43]	Prospective cohort study	Israel	BNT162b2	Haemodialysis patients and controls	557	Diminished and waning immunity to COVID-19 vaccination among haemodialysis patients in Israel: the case for a third vaccine dose
27	Chemaitelly et al. (2021) [44]	Case–control design	Qatar	BNT162b2	General population	907,763	Waning of BNT162b2 Vaccine Protection against SARS-CoV-2 Infection in Qatar

### Data synthesis and presentation

After carefully observing the included studies, we resolved through discussion that the included studies cannot be meta-analysed as the data from the different study designs varied and were not suitable for combining all in a single statistical analysis. Therefore, a narrative synthesis approach was used in organising the data. The data synthesis process paid attention to the fact that the different study designs have different methodological strengths and weaknesses. In other words, similarities and differences in the findings were discussed against the fact that differences in outcomes could occur due to variability in study designs, variability in populations, variability in the interventions, and variability in the study settings. Estimates of the duration or waning period for each vaccine against SARS-CoV-2 were carefully analysed based on data reported in the independent studies. We aimed to synthesise the waning of immunity against SARS-CoV-2 under 95% confidence intervals for all regions and populations for which data were reported. However, the final included studies were largely heterogeneous and disallowed rational data synthesis under 95% confidence intervals. The synthesised data are therefore presented in descriptive formats using Tables.

## Results

### Search output

**Fig. 2:**
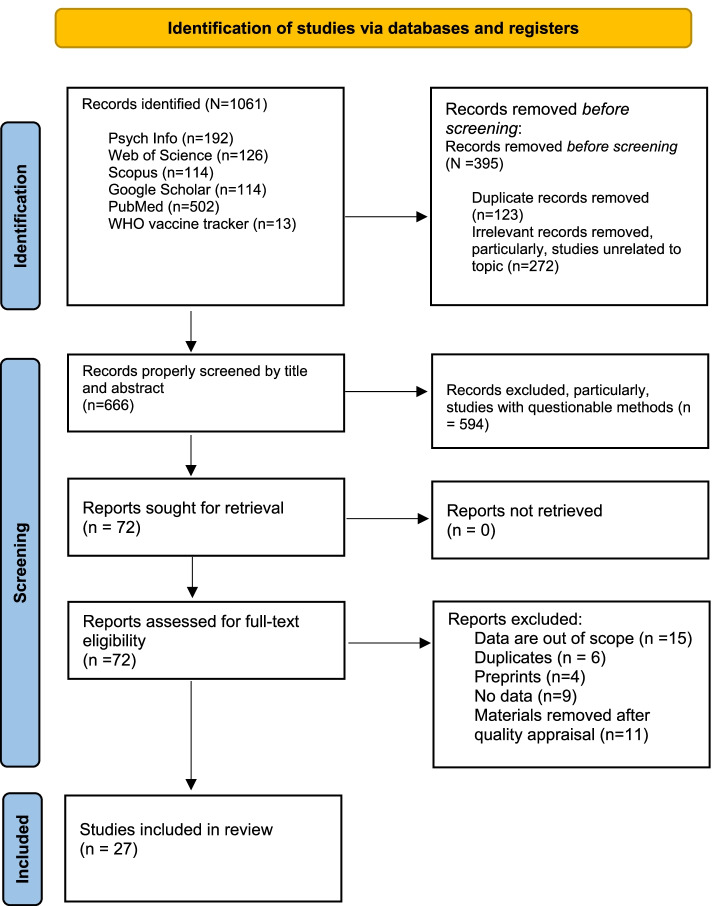
Flow diagram of the review process based on PRISMA 2020 guideline

A total of 1061 studies were identified across the various databases. After removing duplicate records and unrelated studies, 666 studies remained for title and abstract screening. Of the total 666 studies, 72 studies remained for full-text screening as they met the eligibility criteria and were focused on the associations among full dose vaccination against SARS-CoV-2, the impact of full vaccination on immunity against SARS-CoV-2, and/or duration of immunity against SARS-CoV-2. Forty-five out of the 72 studies were excluded after the full-text screening for various reasons: data being out of the study’s scope, studies removed after quality appraisal, studies having duplications (e.g., same studies published with different author arrangements), questionable preprint studies, and studies containing no relevant data. In the end, 27 studies remained for the analysis as shown in Fig. 2.

### Characteristics of the included studies

All the 27 studies included in the final analysis were based on quantitative research designs. The majority were longitudinal studies (*n* = 9) and randomised controlled trials (*n* = 7). Twelve countries were represented, with Israel (*n* = 6), the United Kingdom (*n* = 5), and the United States (*n* = 5) comprising the majority. The number of studies focusing on BNT162b2 was larger than 15 (*n* = 21), however, 15 of those studies focused exclusively on this vaccine. Interestingly, mixed populations were represented in the studies with health workers featuring as the majority (*n* = 9). The combined sample size for the included studies was 16,996,937. Table 2 presents a summary of the included studies.

### Effect of SARS-COV-2 vaccines on immunity

Table 3 presents the direct effect of the vaccines on immunity as reported by the 27 studies fulfilling the inclusion criteria. Of these studies, 24 (88.9%) reported on the immunogenicity and/or the efficacy of the SARS-COV-2 vaccines. Regarding the vaccines’ impact on immunogenicity (serology), one study focusing on BNT162b2 [22] reported that the vaccine influenced an antibody response that reached a maximal level between days 28 and 42 (2204 U/mL versus 1,863 U/mL; *P* = 0.20), while another BNT162b2 study [15] stated that the vaccine influenced high level of anti-SARS-CoV-2 antibody titres (ranging from 0.26 to 14.16, with a mean value of 4.23 ± 2.76) following full vaccination. Further, evidence from other studies focusing primarily on BNT162b2 [21, 26, 29, 30, 33, 38] showed that the vaccine induces substantial antibody levels, resulting in a robust immune response against SARS-CoV-2 infection.

**Table 3:** Direct effects of vaccines and duration of immunity following vaccination as reported by studies fulfilling inclusion criteria

S/n	Study	Vaccine’s direct effect on immunity	Waning or duration of immunity
1	Shrotri et al. (2021) [17]	BNT162b2 increased S-antibody levels to a median of 7506 U/mL (IQR 4925–11 950) at 21–41 days, and ChAdOx1 increased S-antibody levels to a median of 1201 U/mL (IQR 609–1865) at 0–20 days	The authors reported a significant trend of decline in S-antibody levels with time for both AstraZeneca (ChAdOx1) and Pfizer (BNT162b2) vaccines. Specifically, about five-fold and two-fold reductions from peak antibody levels were observed in ChAdOx1 and BNT162b2 respectively, 70 days or more post-second dose vaccination. While S-antibody levels reduced from a median of 7506 U/mL (IQR 4925–11 950) at 21–41 days, to 3320 U/mL (1566–4433) at 70 or more days in the BNT162b2 group, S-antibody levels reduced from a median of 1201 U/mL (IQR 609–1865) at 0–20 days to 190 U/mL (67–644) at 70 or more days in the ChAdOx1 group
2	Shu et al. (2021) [20]	V-01 provoked substantial immune responses in the two-dose group, achieving encouragingly high titres of neutralising antibody and anti-RBD immunoglobulin, which peaked at day 35 (161.9 [95% confidence interval [CI]: 133.3–196.7]	Not applicable
3	Tsatsakis et al. (2021) [15]	The vaccine induced high level of anti-SARS-CoV-2 antibody titres (ranging from 0.26 to 14.16, with a mean value of 4.23 ± 2.76) following complete vaccination	The time of sampling after the second vaccine dose appeared to negatively correlate with antibody titres starting from the third week post-vaccination
4	Levin et al. (2021) [21]	The vaccine induced SARS-CoV-2 antibody with the highest titres (peak) observed during days 4 through 30, after the receipt of the second dose	Six months after receipt of the second dose, neutralising antibody titres were substantially lower among men than among women (ratio of means, 0.64; 95% confidence interval [CI], 0.55 to 0.75), lower among persons 65 years of age or older than among those 18 to less than 45 years of age (ratio of means, 0.58; 95% CI, 0.48 to 0.70), and lower among participants with immunosuppression than among those without immunosuppression (ratio of means, 0.30; 95% CI, 0.20 to 0.46)
5	Favresse et al. (2021) [22]	The vaccine induced antibody response reaching maximal level between days 28 and 42 (2204 versus 1,863; P = 0.20)	A significant antibody decline was observed at 3 months compared to the peak response
6	Taylor et al. (2021) [23]	The vaccines gave a high % neutralising antibodies two weeks after the second dose. Also, the Johnson and Johnson vaccine gave positive neutralising antibody levels after several weeks	At peak concentration, an approximate threefold difference in titer was observed between individuals and all samples exhibited a sharp initial decline in neutralizing antibodies that began to tail off approximately 30 days after the second dose
7	Terpos et al. (2021) [24]	The vaccines induced a median NAb inhibition titre of 62.8% (for patients with myeloma) and 90% for healthy subjects (controls)	Not applicable
8	Flaxman et al. (2021) [25]	ChAdOx1 nCoV-19 induced high antibody titres especially those with longer interval between first and second vaccinations	Not applicable
9	Collier et al. (2021) [26]	The vaccine induced significantly higher geometric mean neutralisation titre (GMT) and increased IgG	Not applicable
10	Ella et al. (2021) [27]	BBV152 induced high neutralising antibody and showed better reactogenicity and safety outcomes, enhancing humoral and cell-mediated immune responses	Not applicable
11	Glockner et al. (2021) [28]	All vaccines induced higher levels of neutralising antibodies in healthy subjects when compared to subjects after a mild infection	At 4–5 weeks of double vaccination, S-IgG levels were 1755 BAU/mL (95% CI: 1219–2527) but at final follow-up (13 weeks), S-IgG levels decreased to 806.6 (95% CI: 598–1087)
12	Goldberg et al. (2021) [29]	The rate of confirmed SARS-CoV-2 infections was significantly lower among the fully vaccinated indicating increased immunity	Findings indicate that immunity against the delta variant of SARS-CoV-2 waned across all age groups a few months post full vaccination
13	Guerrera et al. (2021) [30]	The vaccine induced the development of a sustained anti-viral memory T cell response which includes both the CD4 + and the CD8 + lymphocyte subsets	The vaccine induced antibodies decrease over time, though the antibodies were maintained at high levels for at least 6 months post full vaccination
14	Chu et al. (2021) [31]	The vaccine resulted in significant immune responses to SARS-CoV-2. It induced bAb and nAb by 28 days postvaccination one. Following second vaccination, binding antibodies and nAb increased substantially by 14 days	Not applicable
15	Israel et al. (2021) [32]	Not applicable	The authors reported a gradual increase in the risk of breakthrough infections among participants who received their second vaccine dose after at least 146 days (Please note: Findings from this study should be taken cautiously as it was a preprint at the time of screening).
16	Khoury et al. (2021) [33]	The vaccine induced antibody titres which reached a climate after one month of the second dose of the vaccine	Antibody titre drops rapidly one month after the second dose of the vaccine
17	Frater et al. (2021) [34]	The vaccine induced anti-spike IgG responses by ELISA which peaked at day 42 and were sustained until day 56	Not applicable
18	Nanduri et al. (2021) [35]	Effectiveness estimates showed that the vaccines protect against SARS-CoV-2 infection among nursing home residents	Not applicable
19	Richmond et al. (2021) [36]	SCB-2019 induced immune responses against SARS-CoV-2. It increased the IgG antibodies, ACE2-competitive binding antibodies and neutralising antibodies against SARS-CoV-2	Authors reported that titres waned from their peak at days 36–50, but SCB-2019 IgG antibodies, ACE2-competitive binding antibodies and neutralising antibodies against wild type SARS-CoV-2 persisted at 25–35% of their observed peak levels at Day 184
20	Racine-Brzostek et al. (2021) [37]	The vaccine generated antibody levels similar, if not superior, to the antibody levels induced by natural SARS-CoV-2 infection	The authors reported a decrease in total and neutralising antibodies among participants that were never diagnosed with SARS-CoV-2 four weeks post-second dose vaccination
21	Naaber et al. (2021) [38]	The vaccine induced robust antibody response to Spike protein after the second dose	The antibody levels declined at 12 weeks and 6 months post-vaccination, indicating a waning of the immune response over time
22	Tartof et al. (2021) [39]	The vaccine effectiveness against SARS-CoV-2 infections was 73% (95% CI 72–74) and against COVID-19-related hospital admissions was 90% (89–92)	Vaccine effectiveness against infections declined from 88% (86–89) during the first month after full vaccination to 47% (43–51) after 5 months
23	Tober-Lau et al. (2021) [40]	Not applicable	Significant decline in markers of immunity at the 6-month follow-up, particularly for older participants
24	Achiron et al. (2021) [41]	Not applicable	Anti-S1 IgG levels determined across 1 to 8 months after full vaccination, waned with an estimated half-life of 45 days
25	Aldridge et al. (2021) [42]	Three weeks after the second dose the vaccines induced substantially higher anti-S levels (BNT162b2 mean anti-S levels were 9039 (95%CI: 7946–10,905) U/ml and ChadOx1 were 1025 (95%CI: 917–1146) U/ml)	Waning for both vaccines began three weeks after the second dose. At 20 weeks after the second dose of vaccine, the mean anti-S levels were 1521 (95%CI: 1432–1616) U/ml for BNT162b2 and 342 (95%CI: 322–365) U/ml for ChAdOx1. Further evidence showed that rates of waning were higher in BNT162b2 (- 8.27e-03 [ln (anti-S U/ml)/day], t1⁄2 = 68.81 days) than ChAdOx1 (-10.1e-03 [ln (anti-S U/ml)/day], t1⁄2 = 84.5 days; p < 0.001). No difference in rates of waning was observed by age and sex for both BNT162b2 and ChAdOx1. Also, there was no evidence of a difference in rates of waning by clinical risk groups for both BNT162b2 and ChAdOx1 cohorts (Please note: Findings from this study should be taken cautiously as it was a preprint at the time of screening)
26	Angel-Korman et al. (2021) [43]	The vaccine induced a positive anti-S antibody titre level but was significantly lower in haemodialysis patients than the non-dialysis-dependent	ultivariate analysis demonstrated a gradual antibody waning in MHD patients, with anti-S titres decreasing by 1.36% (95% CI 0.74–1.38%) and neutralising antibodies by 2.37% (1.29–3.63%) per day, as well as loss of neutralising antibodies with time
27	Chemaitelly et al. (2021) [44]	Estimated BNT162b2 effectiveness against any SARS-CoV-2 infection was 77.5% (95% CI, 76.4 to 78.6) in the first month after the second dose. The peak effectiveness against symptomatic infection was 81.5% (95% CI, 79.9 to 83.0), whereas that against asymptomatic infection was 73.1% (95% CI, 70.3 to 75.5)	Estimated BNT162b2 effectiveness against any SARS-CoV-2 infection was negligible for the first 2 weeks after the first dose, increased to 36.8% (95% confidence interval [CI], 33.2 to 40.2) in the third week after the first dose, and reached its peak at 77.5% (95% CI, 76.4 to 78.6) in the first month after the second dose. However, effectiveness declined gradually, starting from the first month after the second dose. The decline accelerated after the fourth month, and effectiveness reached a low level of approximately 20% in months 5 through 7 after the second dose

Studies that focussed on other vaccines reported similar positive effects of the vaccines on immunity. One V-01-based study [20] reported that the V-01 vaccine provoked substantial immune responses reaching high titres of neutralising antibody and anti-RBD immunoglobulin, which peaked at day 35 (161.9 [95% confidence interval [CI]: 133.3–196.7] after two-doses. Flaxman, Marchevsky [25] and Frater, Ewer [34] focused primarily on ChAdOx1 and reported that the vaccine induces high antibody titres following full vaccination. Similarly, Ella, Reddy [27] reported that the BBV152 vaccine induced high neutralising antibodies and showed better reactogenicity that enhanced humoral and cell-mediated immune responses. Evidence from Chu, McPhee [31] also shows that mRNA-1273 vaccine produces significant immune responses to SARS-CoV-2.

Some studies compared the effects of various vaccines on serology. For instance, Shrotri, Navaratnam [17] looked at the BNT162b2 and ChAdOx1 vaccines and reported that BNT162b2 increased S-antibody levels to a median of 7506 U/mL (IQR 4925–11 950) at 21- 41 days, and ChAdOx1 increased S-antibody levels to a median of 1201 U/mL (IQR 609–1865) at 0–20 days. Similarly, Aldridge, Yavlinsky [42] reported on the BNT162b2 and ChAdOx1 vaccines and found that three weeks after the second dose the vaccines induced substantially higher anti-S levels with BNT162b2 inducing mean anti-S levels 9039 (95%CI: 7946–10,905) U/ml and ChadOx1 inducing 1025 (95%CI: 917–1146) U/ml). Further, Glöckner, Hornung [28] focussed on BNT162b2, ChAdOx1, and mRNA-1273 vaccines and highlighted that all the vaccines induced higher levels of neutralising antibodies in participants with no underlying health conditions. Taylor, Hurst [23] also reported that the Ad26.COV2.S, BNT162b2, and mRNA-1273 vaccines stimulated high percentage neutralising antibodies two weeks after full vaccination.

One study which focused on patients with plasma cell neoplasms [24] found that BNT162b2 and ChAdOx1 vaccines induced a less median NAb inhibition titre (62.8%) for the patients with myeloma, compared to 90% for healthy participants. Another study that compared vaccines’ effects on patients receiving haemodialysis and participants with no underlying health conditions [43] reported that though the vaccine (BNT162b2) induced a positive anti-S antibody titre level in persons receiving haemodialysis, the levels were significantly lower than those found in non-dialysis participants. Overall, most of the studies essentially indicated that the vaccines stimulated the production of antibody levels similar, if not superior, to the antibody levels induced by natural SARS-CoV-2 infection.

Concerning the efficacy of the vaccines, one BNT162b2-based study [39] reported that the vaccine’s effectiveness against SARS-CoV-2 infections was 73% (95% CI 72–74) and against COVID-19-related hospitalisation was 90% (89–92). Further, Chemaitelly, Tang [44] reported that the estimated BNT162b2 effectiveness against any SARS-CoV-2 infection was 77.5% (95% CI, 76.4 to 78.6) in the first month after the second dose, with 81.5% (95% CI, 79.9 to 83.0) peak effectiveness against symptomatic infection and 73.1% (95% CI, 70.3 to 75.5) against asymptomatic infection. Generally, the studies reported that the vaccines induced significant immune responses and were effective against SARS-CoV-2 infection.

### Duration of immunity following full vaccination against SARS-CoV-2

Eighteen (66.7%) of the 27 studies included in this systematic review were eligible for assessing the period of waning of vaccine-induced immunity. These 18 studies involved three vaccines: 15 BNT162b2-only studies (83.3%), two BNT162b2 and ChAdOx1 studies (11.1%), and one Clover study (5.6%). Overall, the evidence showed a decline in immunity 3–24 weeks after full vaccination. One study based on BNT162b2 only (15) reported a negative correlation between the time of sampling after the second dose and antibody titre starting from three weeks post-vaccination. Other BNT162b2-based studies have also shown a decline in immunity beginning three months following the second dose [21, 22, 28, 29]. Some studies reported a decline in immunity beginning one month after double vaccination [33, 37, 44], to 6% [33] and 20% [44] of peak immunity levels, four months after the second dose. Further, evidence from one of the included studies showed a decline in immunity from 88% (86–89) during the first month after full vaccination to 47% (43–51) after 5 months [39].

Moreover, evidence from BNT162b2-based studies indicated that there may be a substantial decline in immunity from six months after the second vaccine dose [21, 30, 38, 40, 41]. While waning of immunity specifically against the Delta variant [29] was observed across all age groups [29, 32], other studies have also reported substantially lower neutralising antibody titres or higher waning of immunity six months after the second dose among older than younger people [21, 40], men than women (ratio of means, 0.64; 95% confidence interval [CI], 0.55 to 0.75), and in participants with immunosuppression than among those without immunosuppression (ratio of means, 0.30; 95% CI, 0.20 to 0.46) [21] as well as in patients receiving haemodialysis than in controls [43]. A decline in immunity 6 months after full vaccination has also been reported among participants who received the Clover vaccine [36].

Two studies reported on immunity decline among participant groups who received ChAdOx1 and BNT162b2 vaccines [17, 42]. Shrotri, Navaratnam [17] reported a significant trend of declining S-antibody levels among participants who received BNT162b2 and ChAdOx1 vaccines. At 70 or more days after the second dose, about five- and two-fold decline–compared to peak immunity levels–was respectively observed in ChAdOx1 and BNT162b2 groups; with these levels of decline consistent across age, sex, and clinical vulnerability parameters. While S-antibody levels reduced from a median of 7506 U/mL (IQR 4925–11 950) at 21–41 days, to 3320 U/mL (1566–4433) among the BNT162b2 group at 70 or more days post full vaccination, S-antibody levels reduced from a median of 1201 U/mL (IQR 609–1865) at 0–20 days to 190 U/mL (67–644) among the ChAdOx1 group within the same period. Importantly, 70 or more days post-vaccination, levels of antibody was substantially lower among clinically vulnerable sub-group in the ChAdOx1 cohorts in comparison with the same sub-population in the BNT162b2 cohorts.

Similarly, another study focusing on ChAdOx1 and BNT162b2 cohorts reported anti-S levels means of 9039 (95%CI 7946–10,905) U/ml for BNT162b2 and 1025 (95%CI 917–1146) U/ml for ChAdOx1 three weeks after the second dose. Twenty weeks after the second dose, anti-S levels declined to 1521 (95%CI 1432–1616) U/ml in the BNT162b2 group and 342 (95%CI 322–365) U/ml in the ChAdOx1 group. The study identified 197 breakthrough infections and reported that participants with post full vaccination anti-S levels of 500 U/ml or greater had a reduced risk of breakthrough infection compared with those whose anti-S levels were less than 500 U/ml. Notably, the study also estimated the time to reach an anti-S threshold of 500 U/ml to be 96 days for ChAdOx1 and 257 days for BNT162b2 vaccines. Based on these studies [20, 42], it appears that ChAdOx1 may wane faster than BNT162b2 as the study found that people who received ChAdOx1 were at increased risk of breakthrough infections than those who received BNT162b2 (OR 1.43, 95% CIs1.18–1.73, p < 0.001).

### Risk of bias scores

Overall, most of the included studies were rated low risk in terms of bias whereas three studies were rated high risk (Fig. 1). Nevertheless, the study by [24] had a limited number of patients in the subgroup analyses including the absence of data on T-cell induced immune responses following vaccination against SARS-CoV-2. Some studies, such as [27], reported interim results from phase 2 trials with possibilities of new outcomes in a phase 3 trial and hence should be taken with caution. Furthermore, a few studies [32, 42] were yet to undergo peer preview and should also be taken carefully. Additionally, the threshold for measuring vaccine-induced immunity was inconsistent across the included studies and was implied in some studies. We also observed that most of the included studies confounded the effect of differences in natural immunity against SARS-CoV-2 on the duration of vaccine-induced immunity as it seemed to be difficult to separate. The units for some reported values were missing in some studies, for instance, the antibody titres reported by [15], making interpretation of the results quite complicated.

## Discussion

Vaccination against SARS-CoV-2 undisputedly protects against severe illnesses, hospitalisations, and deaths from SARS-CoV-2 infections [7, 8]. However, the new surge in SARS-CoV-2 infections, due to the highly transmissible Delta and Omicron variants, may likely retard progress that has been attained in reducing the disruptions that the pandemic has caused in almost every facet of human life [3]. This surge also includes re-infections in people that have been fully vaccinated against SARS-CoV-2 [2], thereby raising serious concerns about the duration of the potency of immunity resulting from vaccination.

The overall findings demonstrate that all the vaccines reported in the studies included in this systematic review, successfully stimulated the production of significant antibody levels, resulting in a robust immune response against SARS-CoV-2 infections. This finding is consistent with several previous studies that considered the immunogenicity or efficacy of SARS-CoV-2 vaccines [45–47]. Further, the analysis showed that the vaccine-induced protection against SARS-CoV-2 infection builds rapidly after a person receives the first dose of the vaccines and peaks within 4 to 42 days after the second dose, before gradually starting to wane in subsequent months, usually from 3 to 24 weeks. This finding implies that administering booster doses between 3 to 24 weeks following full vaccination will be important in maintaining optimal protection against SARS-COV-2 infections. However, the finding also begs the question as to whether it is sustainable, equitable, and cost-effective to continue administering vaccine boosters over time. The importance of administering booster doses in the future remains debatable considering that a large proportion of the world population remains unvaccinated and does not have access to vaccines for various reasons [4].

It is also important to note that there were reported variations in the duration of vaccine-induced immunity against SARS-CoV-2 infections across the different vaccine types and population parameters. For instance, vaccine-induced antibody response levels were higher in people with no reported underlying health conditions compared to those with immunosuppressed conditions, such as people receiving haemodialysis and those living with myeloma. The decline in immunity following full vaccination was also reported to be higher among elderly participants and men than their respective counterparts. However, this finding should be interpreted cautiously given the limited amount of evidence that was found. Reports from two studies [20, 42] also indicate potential differences in the timing of the reduction in protection induced by SARS-COV-2 vaccines. Nevertheless, these findings should be taken cautiously as more than half of the participants received BNT162b2 vaccines with a relatively small proportion receiving other vaccine types. Overall, these findings point to the need to rethink the “one-size-fits-all” approach in the administration of vaccines and follow-up boosters. The evidence brings to attention the need to prioritise vulnerable populations in booster administration exercises.

### Limitations

This systematic review was conducted in accordance with international protocol guidelines for conducting a systematic search for relevant studies, assessing methodological quality, and synthesising results. However, the findings could still be subjected to indexing, publication, and reporting bias as the scope of the search was limited to Psych Info, Web of Science, Scopus, Google Scholar, PubMed, and WHO COVID-19 database. Also, only studies published in English were included and this means that eligible studies in other languages may be missed, resulting in a potential selection bias. Moreover, this review could be limited in scope as only research papers published online were included, considering the fact that not all case studies, cohort studies, and clinical trial outcomes are published online. Furthermore, quantitative synthesis could not be done due to wide methodological and data variations among the included studies. Additionally, BNT162b2 (n = 15) predominated the vaccine types making the comparison of reports on the different vaccines quite superfluous and difficult. Our narrative approach to the data syntheses may also be prone to data interpretation bias and errors. However, the specific sources of included studies are presented in the included Tables for easy referencing. Also, we mainly evaluated the immune response rate after the administration of the second dose for two-dosage vaccines, which can be contestable given that booster doses are already being rolled-out. Additionally, the duration of immunity may be affected by differences in SARS-CoV-2 variants and variations in the levels of SARS-CoV-2 circulation, severity, and virulence. These two important indicators are not reported in this review as the study was conceptualised before the emergence of the Omicron variant and its numerous sub-lineages. Therefore, the included studies did not explicitly cover the differential impact of different SARS-CoV-2 variants on the duration of vaccine-induced immunity. Lastly, the measurement of the duration of immunity following full vaccination as reported in some of the studies may be confounded by differences in natural immunity and variations in immunity induced by previous infections which are difficult to establish as well as differences in population characteristics.

## Conclusions

This systematic review shows that although the SARS-CoV-2 vaccines induce protection against the virus, this protection wanes over time thereby necessitating booster doses. The waning of vaccine-induced protection against SARS-CoV-2 usually begins from 3 to 24 weeks after receiving a full dose. The study also demonstrates that vaccine-induced antibody response levels vary across different populations and seem to be higher in people with no underlying health conditions compared to those with immunosuppressed conditions, such as people receiving haemodialysis and those living with myeloma. It was also found that there were variations in the duration of vaccine-induced immunity across vaccine types, however the supporting evidence was not very strong and therefore should be taken cautiously. While the evidence synthesised in this review could effectively inform vaccine booster policies, we believe that more studies, especially clinical trials, are still needed to ascertain the exact duration of immunity, especially across different population and vulnerability groups.

## Data Availability

All-important data generated or analysed during this study are included in this article. The other materials describing the various stages of the study are available from the corresponding author on reasonable request.

## Abbreviations

COVID-19: Corona Virus Disease 2019
JBI: Joana Briggs Institute
MEDLINE: Medical Literature Analysis and Retrieval System Online
PROSPERO: Prospective Register of Systematic Reviews
PsycINFO: Psychological Information Database
PubMed: Public/Publisher MEDLINE
RoB 2: Risk of Bias tool—version 2
SARS-CoV-2: Severe Acute Respiratory Syndrome Coronavirus 2
SWiM: Synthesis Without Meta-analysis
